# PD-L1 expression on peripheral T-cells and association with coronary heart disease patients: A protocol for systematic reviews and meta-analysis

**DOI:** 10.1097/MD.0000000000025157

**Published:** 2021-03-26

**Authors:** Chunwei Zhang, Ke Yang, Ying Yang, Gang Zhao

**Affiliations:** a Department of cardiovascular, Clinical Medical College &Afiliated Hospital of Chengdu University, Chengdu University, Chengdu, Sichuan Province; b Department of cardiovascular, University-Town Hospital of Chongqing Medical University, Chongqing, China.

**Keywords:** coronary heart disease, meta-analysis, peripheral T-cells, programmed death ligand-1, protocol

## Abstract

**Background:**

As immune checkpoint pathways, programmed death-1 (PD-1) and programmed death ligand-1 (PD-L1) can be exploited by tumor cells to evade immuno-surveillance. Inflammation and immune processes play decisive roles in the occurrence and development of coronary heart disease (CHD). The low expression level of PD-1/ PD-L1 or anti-PD-1/PD-L1 therapy can accelerate the immune processes in CHD and aggravates disease based on numerous studies. However, the expression of PD-L1 and CHD still remains controversial to date. We conducted this meta-analysis to detect the value of PD-L1 expression on peripheral T-cells in CHD.

**Methods:**

We will search PubMed, Embase, Web of Science, Google Scholar, Chinese National Knowledge Infrastructure, Chinese VIP Information, Wanfang Database, and Chinese Biomedical Literature Database for related published studies before February 2021. Two review authors will search and assess relevant studies independently. Case control studies and cohort studies will be included. The Revman 5.3 software was applied to carry out the meta-analysis for the included literature.

**Results:**

The findings of this systematic review will be disseminated in a peer-reviewed publication and/or presented at relevant conferences.

**Conclusion:**

This study will provide a new theoretical basis for the immunological prevention and treatment of CHD.

**Trial registration number:**

DOI 10.17605/OSF.IO/X3R52.

**Ethics and dissemination:**

Formal ethical approval is not required, as the data are not individualized.

## 1 Introduction

As the most common cardiovascular disease, coronary heart disease (CHD) is the leading cause of death and disability in developed/developing countries.^[1–3]^ Despite the development of treatment over the past decade, 1/3 of deaths among people over the age of 35 are still caused by the disease.^[4–6]^ In 2014, the US Centers for Disease Control and Prevention reported that the prevalence rate of CHD, angina pectoris and myocardial infarction among US and Asian residents were 6.0%, 1.9%, 3.2% and 2.8%, 0.8%, and 1.4%, respectively.^[7]^

CHD is a complex disease that is caused by various factors, including smoking, drinking, hypertension, diabetes, genetics, environment, occupation, age, sex, race, dyslipidemia, autoimmune diseases, rheumatoid diseases, obesity, lifestyle and others.^[8,9]^ These factors affect CHD through different mechanisms. Due to complex risk factors and individual differences, the prognosis of patients with CHD is different, so individualized treatment is adopted. Common treatments include hypolipidemic drugs, anticoagulants, β-adrenoceptor inhibitors, heart rate control drugs and strategies for primary diseases. All of these treatments are aimed at improving coronary artery stenosis and plaque stability, so as to prevent acute coronary syndrome, including acute myocardial infarction, angina and death. It is generally believed that CHD is regulated by the immune process.^[10,11]^ In recent years, more and more studies have revealed that inflammation and immune response play important roles in the occurrence and development. The study of immunological pathogenesis and immunological intervention of CHD has attracted attentions of researchers.

The activation of programmed death receptor-1/programmed death receptor-1 ligand-1 (PD-L1) signaling pathway widely participates in a series of processes such as T cell activation, proliferation and apoptosis, and inhibits T cell-mediated cellular immune response.^[12–14]^ With the deepening of its basic research, it is also considered to be closely related to different cardiovascular diseases.^[15–18]^ PD-L1 molecule is a member of B7 family, and is widely expressed in lymphocytes, other peripheral tissues and cells.^[19,20]^ PD-L1 mainly binds to programmed death-1 and expresses negative regulation, which suppresses the immune response in vivo, delays the occurrence of atherosclerosis and inhibits various inflammatory factors.^[21]^

Many studies have reported the relationship between the expression of PD-L1 in peripheral blood T lymphocytes and CHD.^[21–23]^ However, the results of these studies are not consistent. Therefore, we performed meta-analysis to examine the accurate correlation between the expression of PD-L1 in peripheral blood T lymphocytes and CHD.

### 1.1 Protocol register

This protocol of systematic review and meta-analysis has been drafted under the guidance of the preferred reporting items for systematic reviews and meta-analysis protocols.^[24]^ Moreover, it has been registered on the OSF (registration number: DOI 10.17605/OSF.IO/X3R52).

### 1.2 Ethics

Since this is a protocol without patient recruitment and personal information collection, approval by the ethics committee is not required.

### 1.3 Eligibility criteria

Articles were included if they met the following criteria:

1) Case-control, or cohort studies.
2) CHD patients were diagnosed by coronary angiogram.
3) Control subjects were people without the history of any autoimmune disorders including CHD.
4) Results contained the evaluation of PD-L1 expression on peripheral T-cells.
5) Language would be restricted to Chinese and English.

### 1.4 Exclusion criteria

1) Animal-model studies, case reports, review articles, letters, comments, and editorials.
2) The published papers were abstracts or the data were incomplete, and the papers with complete data were not available after contacting the author.
3) Papers containing less than 10 cases.

### 1.5 Information sources and search strategy

The search will use a sensitive subject and topic-based strategy from inception to February 2021. The searched database includes PubMed, Embase, Web of Science, Google Scholar, Chinese National Knowledge Infrastructure, Chinese VIP Information, Wanfang Database, and Chinese Biomedical Literature Database. Taking PubMed as an example, the retrieval strategy is demonstrated in Table 1.

**Table 1:** Search strategy for the PubMed database.

Number	Search terms
#1	Coronary Disease[MeSH]
#2	Coronary Heart Disease[Title/Abstract]
#3	Coronary Diseases[Title/Abstract]
#4	Coronary Heart Diseases[Title/Abstract]
#5	Disease, Coronary[Title/Abstract]
#6	Disease, Coronary Heart[Title/Abstract]
#7	Diseases, Coronary[Title/Abstract]
#8	Diseases, Coronary Heart[Title/Abstract]
#9	Heart Disease, Coronary[Title/Abstract]
#10	Heart Diseases, Coronary[Title/Abstract]
#11	Coronary Artery Disease[MeSH]
#12	Arteriosclerosis, Coronary[Title/Abstract]
#13	Atherosclerosis, Coronary[Title/Abstract]
#14	Coronary Arteriosclerosis[Title/Abstract]
#15	Coronary Atherosclerosis[Title/Abstract]
#16	Arterioscleroses, Coronary[Title/Abstract]
#17	Artery Disease, Coronary[Title/Abstract]
#18	Artery Diseases, Coronary[Title/Abstract]
#19	Atheroscleroses, Coronary[Title/Abstract]
#20	Coronary Arterioscleroses[Title/Abstract]
#21	Coronary Artery Diseases[Title/Abstract]
#22	Coronary Atheroscleroses[Title/Abstract]
#23	Disease, Coronary Artery[Title/Abstract]
#24	Diseases, Coronary Artery[Title/Abstract]
#25	or/1–24
#26	Programmed death ligand-1[Title/Abstract]
#27	PD-L1[Title/Abstract]
#28	or/26–27
#29	#25 and #28

PD-L1 = programmed death ligand-1.

### 1.6 Data filtering and extraction

**Figure 1:**
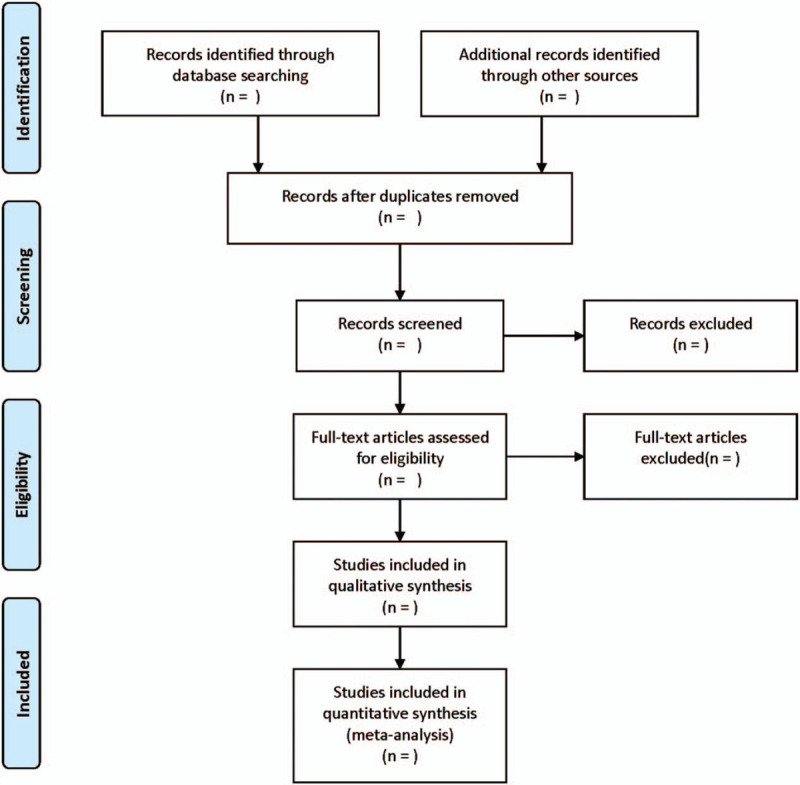
The process of literature filtering.

Data extraction and quality evaluation were performed independently by 2 researchers. In case of missing data in the included studies, the corresponding author was emailed to obtain additional information or raw data. Any disagreements between the 2 researchers were resolved through discussion. The researchers record the reasons to exclude each study in light of the preferred reporting items for systematic reviews and meta-analysis guidelines and report the screening results. After screening, we tried to extract the basic information [e.g., first author, year, region, ethnicity, sample source, sample size, mean value, standard deviation (SD) value, assay, etc.] from the selected full-text articles. The process of literature filtering is exhibited in Figure 1.

### 1.7 Literature quality assessment

As the studies involved in this meta-analysis are all case-control ones, the quality of the included studies was assessed by Newcastle-Ottawa Scale.^[25]^ In this scale, “patient selection,” “comparability of study groups” and “exposure” consist of a particular “star system” to evaluate the included studies. The lowest score was 0 star, and the highest score was 9 stars. Studies, with the score ≥5 stars, were defined as high quality. On the other hand, studies, with a score <5 stars, were defined as low quality. The quality of all included studies was assessed by 2 authors. Discrepancy was resolved through the discussion between the 2 authors.

### 1.8 Statistical analysis

#### 1.8.1 Data analysis and processing

Only when enough and suitable data were harvested, a meta-analysis was conducted using RevMan 5.3. In the Cohen statistics of our association test, *P* (*P* value of association), SMD (standard mean difference), and 95%CI (confidence intervals) were calculated. Heterogeneity was ascertained using *I*^2^. *I*^2^ < 50% revealed that the studies exhibited homogeneity, so fixed effects model was used. Otherwise, the random effects model was adopted. In the presence of heterogeneity, sensitivity analyses and subgroup analysis would be conducted to investigate heterogeneity sources.

#### 1.8.2 Dealing with missing data

If there are missing data in the article, the author would be contacted via email for additional information. If the author cannot be contacted, or the author has lost relevant data, descriptive analysis will be conducted instead of meta-analysis.

#### 1.8.3 Sensitivity analysis

In order to test the stability of meta-analysis results of indicators, a one-by-one elimination method will be adopted for sensitivity analysis.

#### 1.8.4 Assessment of reporting biases

Publication bias was assessed by funnel plot that was performed for no less than 10 studies.^[26,27]^

#### 1.8.5 Subgroup analysis

We performed the subgroup analyses by factors of ethnicity (Caucasian/Asian) and control source [PB (population-based control)/HB (hospital-based control)].

## 2 Discussion

CHD is the most common chronic disease with the highest mortality in the world, and the process of its occurrence and development is always accompanied by the aggravation of atherosclerosis.^[28,29]^ Studies have proved that PD-L is involved in the pathogenesis of atherosclerosis through immunity.^[30–32]^ Many scholars have investigated the relationship between the expression of PD-L1 in peripheral blood T lymphocytes and CHD, but the results are different and the sample size is small. This study will be the first meta-analysis to investigate the current available evidence to identify the association between the expression of PD-L1 in peripheral blood T lymphocytes and CHD. It is expected that the results of this meta-analysis can inform the relationship between the expression of PD-L1 in peripheral blood T lymphocytes and CHD, which may be beneficial to clinical practices and further researches.

The advantages of this study include the following aspects: We will include the latest literature. As for the exploration of heterogeneity, we will try to avoid post-group subgroup analysis. In order to improve the credibility of the results, we will carry out sensitivity analysis.

In conclusion, our study will provide evidence for the association between the expression of PD-L1 in peripheral blood T lymphocytes and CHD. These findings may offer new insights into treatment strategies to reduce the risk of CHD in clinical environment.

## Author contributions

**Conceptualization**: Chunwei Zhang and Gang Zhao.

**Data curation**: Chunwei Zhang and Ke Yang.

**Formal analysis:** Chunwei Zhang.

**Funding acquisition**: Gang Zhao.

**Investigation:** Chunwei Zhang, Ying Yang.

**Methodology**: Ying Yang.

**Project administration**: Gang Zhao.

**Resources:** Ke Yang, Ying Yang.

**Software:** Ke Yang, Ying Yang.

**Supervision**: Chunwei Zhang and Ke Yang, Ying Yang.

**Validation**: Chunwei Zhang and Ke Yang, Ying Yang.

**Visualization:** Ke Yang, Ying Yang.

**Writing – original draft**: Chunwei Zhang and Gang Zhao.

**Writing – review & editing**: Chunwei Zhang and Gang Zhao.
